# Clinical potential of [^18^F]FET PET in patients with circumscribed astrocytic glioma

**DOI:** 10.1007/s00259-025-07654-9

**Published:** 2025-11-18

**Authors:** Jan-Michael Werner, Maximilian J. Mair, Michael M. Wollring, Enio Barci, Isabelle Stetter, Hannah C. Puhr, Caroline Tscherpel, Gabriele Stoffels, Johannes A. Hainfellner, Anna S. Berghoff, Vincent Sunder-Plassmann, Georg Widhalm, Franziska Eckert, Gregor Kasprian, Thomas S. Nakuz, Alexander Beck, Patrick N. Harter, Louisa von Baumgarten, Niklas Thon, Stephan Schönecker, Robert Forbrig, Felix M. Mottaghy, Philipp Lohmann, Gereon R. Fink, Karl-Josef Langen, Norbert Galldiks, Nathalie L. Albert, Matthias Preusser

**Affiliations:** 1 Division of Oncology, Department of Medicine I, Medical University of Vienna, Vienna, Austria; 2 Christian Doppler Laboratory for Personalized Immunotherapy, Department of Medicine I, Medical University of Vienna, Vienna, Austria; 3 Department of Neurology, Faculty of Medicine and University Hospital Cologne, University of Cologne, Cologne, Germany; 4 Department of Nuclear Medicine, LMU University Hospital, LMU Munich, Munich, Germany; 5 Department of Neurology, University Hospital Frankfurt, Goethe University, Frankfurt am Main, Germany; 6 Institute of Neuroscience and Medicine (INM-3, INM-4), Research Center Juelich, Juelich, Germany; 7 Division of Neuropathology and Neurochemistry, Department of Neurology, Medical University of Vienna, Vienna, Austria; 8 Department of Neurosurgery, Medical University of Vienna, Vienna, Austria; 9 Department of Radiation Oncology, Comprehensive Cancer Center Vienna, Medical University of Vienna, Vienna, Austria; 10 Department of Biomedical Imaging and Image-Guided Therapy, Medical University of Vienna, Vienna, Austria; 11 Division of Neuroradiology and Musculoskeletal Radiology, Medical University of Vienna, Vienna, Austria; 12 Division of Nuclear Medicine, Medical University of Vienna, Vienna, Austria; 13 Center for Neuropathology and Prion Research, LMU University Hospital, LMU Munich, Munich, Germany; 14 German Cancer Consortium (DKTK), University Hospital, Partnersite LMU Munich, Munich, Germany; 15 Bavarian Cancer Research Center (BZKF), Munich, Germany; 16 Department of Neurology, LMU University Hospital, LMU Munich, Munich, Germany; 17 Department of Neurosurgery, LMU University Hospital, LMU Munich, Munich, Germany; 18 Department of Neurosurgery, Knappschaft University Hospital Bochum, Bochum, Germany; 19 Department Radiation Oncology, LMU University Hospital, LMU Munich, Munich, Germany; 20 Institute of Neuroradiology, LMU University Hospital, LMU Munich, Munich, Germany; 21 Department of Nuclear Medicine, University Hospital RWTH Aachen, Aachen, Germany; 22 Department of Radiology and Nuclear Medicine, Maastricht University Medical Center (MUMC+), Maastricht, The Netherlands; 23 Center for Integrated Oncology Aachen Bonn Cologne Duesseldorf (CIO ABCD), Aachen, Germany

**Keywords:** PET RANO 1.0, Response assessment, BRAF, Treatment-related changes

## Abstract

**Purpose:**

To investigate the clinical potential of *O*-(2-[^18^F]fluoroethyl)-L-tyrosine ([^18^F]FET) PET imaging in the management of circumscribed astrocytic gliomas (CAG), a rare glioma subtype with limited imaging data.

**Methods:**

We retrospectively identified adult CAG patients who underwent [^18^F]FET PET imaging (i) before diagnosis, (ii) at suspected relapse, or (iii) for response assessment at three institutions. Maximum and mean tumor-to-brain ratios (TBR_max_, TBR_mean_) and metabolic tumor volumes were assessed according to the PET RANO 1.0 criteria. Diagnostic performance in differentiating treatment-related changes from tumor relapse was evaluated using ROC analysis and Fisher’s exact test.

**Results:**

We evaluated 79 [^18^F]FET PET scans of 42 patients, including nine (21%) with actionable molecular targets. Measurable [^18^F]FET uptake was observed in 65% of WHO grade 1 and 100% of WHO grade 2 and 3 CAG. TBR values were significantly higher in WHO grade 2 and 3 CAG than in pilocytic astrocytomas (*P* < 0.01), but showed no difference based on molecular target status (*P* > 0.05). In 5 of 11 patients (45%), treatment response assessment by PET RANO 1.0 differed from MRI. Treatment-related changes were confirmed in 12 patients (43%). In CAG WHO grades 2 or 3, the accuracy of [^18^F]FET PET to identify treatment-related changes was 82% using single PET scans and 100% using serial PET imaging (*P* < 0.05).

**Conclusions:**

[^18^F]FET PET can contribute to clinical management of patients with CAG by detecting measurable disease, differentiating treatment-related changes from tumor progression, and showing potential in treatment response assessment through longitudinal imaging.

**Supplementary Information:**

The online version contains supplementary material available at 10.1007/s00259-025-07654-9.

## Introduction

Gliomas are the most common malignant primary brain tumors in adults. The latest edition of the WHO classification of tumors of the Central Nervous System (WHO CNS5) classifies these into the families of *diffuse* and *circumscribed* gliomas [1]. The circumscribed astrocytic gliomas (CAG) group demonstrates a more solid growth pattern, whereas diffuse gliomas are characterized by infiltrative and invasive growth. Included in the group of CAG are pilocytic astrocytoma (PA), high-grade astrocytoma with piloid features (HGAP), pleomorphic xanthoastrocytoma (PXA), subependymal giant cell astrocytoma (SEGA), chordoid glioma, and MN1-altered astroblastoma (AB) [1]. Recommended treatment options include gross-total resection, re-resections in case of local relapse, radiotherapy following subtotal resection of CNS WHO grade 2 or CNS WHO 3 CAG, and radiosurgery for small tumor relapses [2]. Especially in PXA, PA, and glioneuronal tumors such as ganglioglioma, somatic alterations in the *BRAF* gene (BRAF^V600E^ mutation and BRAF fusions) represent targets for BRAF and/or MEK inhibitors [2–5]. Based on efficacy data from prospective trials, including PXA and ganglioglioma, BRAF inhibition is recommended in BRAF^V600E^ mutant CNS tumors according to current guidelines [6–9].

Common neuroimaging features of CAG on contrast-enhanced MRI include solid and cystic masses, enhancing nodules (PA, PXA), an enhancing dural tail (PXA), inhomogeneous signal changes with rim enhancement (HGAP, AB), and typical locations (SEGA, lateral ventricle adjacent to the foramen of Monro; chordoid glioma, third ventricle) [2, 10]. Due to the rarity and recent redefinition of the diagnoses, few imaging studies of CAG exist, and some of the reported data are anecdotal. Despite its excellent spatial resolution, conventional MRI suffers from low specificity, and the differentiation of actual brain tumor relapse from treatment-related changes related to novel therapeutic options like targeted treatments remains challenging [11, 12]. Treatment-related changes following BRAF inhibition have been reported in pediatric gliomas and may also occur in adults [13].

As an adjunct to MRI, PET using amino acid tracers has evolved in the clinical management of gliomas, providing complementary information, particularly in the differentiation between treatment-related changes and tumor relapse, and for response assessment [11, 14, 15]. Until now, amino acid PET studies in glioma focused on diffuse gliomas, predominantly glioblastoma [15–18]. The few available studies on evaluating CAG using amino acid PET are based on case-reports [19, 20]. Consequently, the recently published PET-based response assessment criteria (PET RANO 1.0) of the Response Assessment in Neuro-Oncology (RANO) group is limited to diffuse gliomas [21]. However, the higher rate of actionable targets in CAG compared to diffuse gliomas and resulting treatment options make accurate identification of response and relapse pivotal in clinical practice and trials. Therefore, the purpose of this retrospective multicenter study was to investigate the clinical potential of amino acid PET using *O*-(2-[^18^F]-fluoroethyl)-L-tyrosine ([^18^F]F-FET) in patients with CAG.

## Patients and methods

### Patients

We retrospectively assessed data from 42 adult patients with neuropathologically confirmed CAG. All patients underwent [^18^F]FET PET imaging either (i) before diagnosis, (ii) at suspected relapse, or (iii) for response assessment at three institutions (Medical University of Vienna, Research Center Juelich, and LMU Munich). Patients were not stratified for age or sex. Diagnoses of the patients were based on the fifth edition of the WHO Classification of Tumors of the Central Nervous System (WHO CNS) [1], including neuropathological re-evaluation of patients diagnosed before 2021. Testing for actionable molecular targets was performed as requested by the respective treating centers, with variations in the methods used due to the initial diagnoses made between 1992 and 2024. Molecular targets with ESMO Scale for Clinical Actionability of Molecular Targets (ESCAT) evidence tier I-III were considered actionable. Testing for actionable targets was no prespecified endpoint and used to better characterize the patients and inform on all patient-related data available. The extent of surgical resection was obtained from early postoperative MR imaging within 48 h after surgery.

### PET imaging and PET data analysis

PET imaging was performed either at the Division of Nuclear Medicine, Medical University of Vienna, Austria (*n* = 9 patients), the Department of Nuclear Medicine of the RWTH Aachen University, Germany, embedded within the Institute of Neuroscience and Medicine at the Research Center Juelich, Germany (*n* = 19 patients), or the Department of Nuclear Medicine, LMU Munich, Germany (*n* = 14 patients). PET data were analyzed according to PET RANO 1.0 criteria [21], using metabolic tumor volumes and tumor-to-brain ratio (TBR_max_ and TBR_mean_), along with the corresponding classifications for *measurable disease* and *PET-based response* for based on changes in [^18^F]FET uptake. The confirmation of tumor relapse was based on neuropathological analysis (i.e., presence of viable tumor tissue) after repeated biopsy or resection or confirmed clinicoradiologically if a neuropathological diagnosis was unavailable. Further details are provided in the Supplemental Methods (Online Resource 1).

### Statistical evaluation

Descriptive statistics are provided as mean and standard deviation and/or median and range. For group comparison, the two-sided Student t-test was used for independent samples, and the Mann–Whitney rank-sum test was used when variables were not normally distributed. The TBR was considered as 1.0 in patients with no measurable disease. The diagnostic performance for identifying treatment-related changes was assessed by receiver operating characteristic (ROC) curve analyses using the optimal cut-off as the point maximizing the product of sensitivity and specificity. The area under the ROC curve (AUC) and its standard error were calculated, and the diagnostic performance of TBRs was evaluated using the Fisher exact test for 2 × 2 contingency tables. *P*-values of 0.05 or less were considered significant. Given the hypothesis-generating design of the study, no correction for multiple testing was performed [22]. Statistical analyses were performed using GraphPad Prism (RRID:SCR_002798; release 10.3.0, GraphPad Software Inc., La Jolla, CA, USA).

## Results

### Patients

**Fig. 1:**
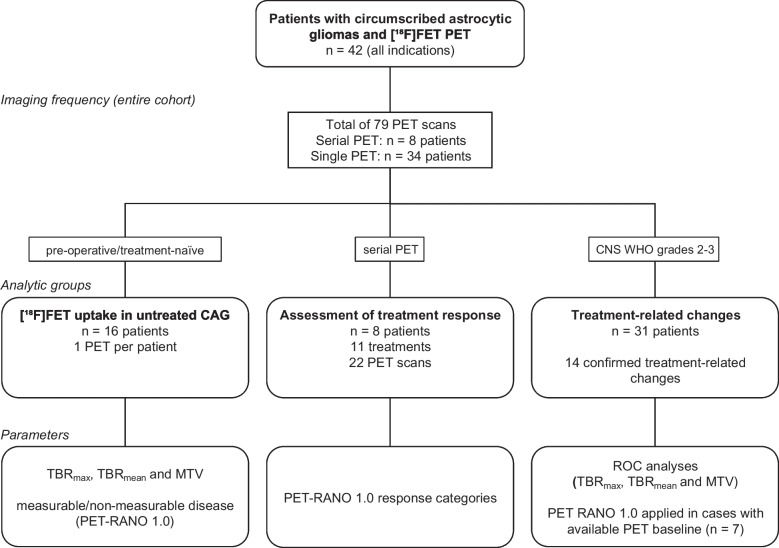
Flow diagram of cohort assembly for [^18^F]FET PET in patients with circumscribed astrocytic gliomas (CAG) and the parameters evaluated. Abbreviations: MTV = metabolic tumor volume; TBR = tumor-to-brain ratio

By diagnostic criteria, the CAG in the patient cohort studied consisted of 25 PA, four PXA CNS WHO grade 2, five PXA CNS WHO grade 3, one AB, and seven HGAP (median age at first PET scan, 35 years; range, 18—73 years; 55% female). Four patients (10%) were diagnosed with neurofibromatosis type 1, and 15 patients (36%) had actionable molecular targets (ESCAT evidence tier I-III). In 42 patients with CAG, 79 [^18^F]FET PET scans were available for evaluation (median number of PET scans, 1; range of PET scans, 1–17). Eleven patients received more than one PET scan. The most frequent indication for [^18^F]FET PET imaging was the differentiation between relapse and treatment-related changes (24 patients, 57%), followed by diagnostic PET for differential diagnosis before the initial neuropathological diagnosis (16 patients, 38%) and response assessment (8 patients, 19%). Patient characteristics, along with the number and indications for [^18^F]FET PET scans, are summarized in Table 1 and detailed for each patient in Supplemental Table 1 (Online Resource 2). Figure 1 illustrates the assembly of the analysis cohorts in a flowchart and Supplemental Fig. 1 (Online Resource 3) presents a swimmer plot of eight exemplary patients with CAG, highlighting the complexity and diversity of their treatments during their long course of disease. It includes multiple [^18^F]FET PET scans used for identifying tumor relapse and/or assessing treatment response, alongside follow-up and treatment data.

**Table 1:** Characteristics of patients with circumscribed astrocytic glioma and [^18^F]FET PET imaging

Characteristic	*n*	%
Number of PET scans per patient		
Median number	1 (range, 1–17)	
1 PET scan	30	71%
2–3 PET scans	7	17%
> 3 PET scans	5	12%
Median age at first PET (years)	35 (range, 18–73)	
Sex		
Female	23	55%
Male	19	45%
Diagnosis (all patients, *N* = 42)		
PA, CNS WHO grade 1	25	60%
PXA, CNS WHO grade 2	4	10%
PXA, CNS WHO grade 3	5	12%
AB *MN1*-altered, CNS WHO grade 3	1	2%
HGAP	7	17%
Molecular targets ESCAT I-III		
BRAF^V600E^	7	17%
KIAA1549::BRAF	2	5%
PDGFRA	2	5%
NF1	5	12%
No target	21	50%
Not tested	6	14%
Neurofibromatosis type 1 (germline mutation)	4	10%
Extent of initial resection		
Gross-total resection	9	21%
Subtotal resection	8	19%
Biopsy	20	48%
No information on the extent	5	12%
PET scans before neuropathological diagnosis (16 patients)		
PA, CNS WHO grade 1	12	75%
PXA, CNS WHO grade 2	1	6
HGAP	3	19%
Identification of treatment-related changes (24 patients)		
Number of PET scans	32	
Diagnosis of treatment-related changes	14	45%
Median number of treatments before PET scan	1 (range, 1–7)	
Assessment of treatment response (8 patients)		
Number of monitored treatments	11	
Alkylating chemotherapy	4	36%
Iodine-125 brachytherapy	3	27%
Targeted therapy	3	27%
Bevacizumab	1	9%

Abbreviations:* AB* astroblastoma, *APA* anaplastic pilocytic astrocytoma, *B* biopsy, *ESCAT* ESMO Scale for Clinical Actionability of Molecular Targets, *GTR* gross-total resection, *HGAP* high-grade astrocytoma with piloid features, *NF1* neurofibromin 1, *PA* pilocytic astrocytoma, *PDGFRA* platelet-derived growth factor receptor A, *PXA* pleomorphic xanthoastrocytoma

### [^18^F]FET uptake in circumscribed astrocytic gliomas

The uptake of [^18^F]FET was evaluated in patients with (i) untreated CAG before initial resection or biopsy, (ii) at confirmed relapse, or (iii) at baseline before initiation of therapy (*n* = 31). In patients with multiple PET scans/lesions, the scan/lesion with the highest lesional uptake, as assessed by TBR values, was considered. According to the PET RANO 1.0 criteria [21], [^18^F]FET PET scans showed *measurable disease* in 77% of CAG (24 out of 31 patients). Moreover, all patients (100%) diagnosed with CAG CNS WHO grades 2 or 3 presented with *measurable disease*. In contrast, in patients with PA CNS WHO grade 1, the rate of *measurable disease* at any point during the disease course was 65% (11 out of 17 patients with PA). TBR_max_ and TBR_mean_ of [^18^F]FET uptake in CAG were significantly higher in CAG CNS WHO grades 2 or 3 compared to PA CNS WHO grade 1 (TBR_max_, 3.8 ± 1.3 vs. 2.3 ± 1.0, *P* < 0.001; TBR_mean_, 2.2 ± 0.3 vs. 1.7 ± 0.4, *P* = 0.001). There was no significant difference in mean or maximum TBR values between CAG with or without actionable targets (TBR_max_, 2.9 ± 1.3 vs. 3.1 ± 1.4, *P* = 0.735; TBR_mean_, 1.9 ± 0.4 vs. 2.0 ± 0.5, *P* = 0.697).

**Fig. 2:**
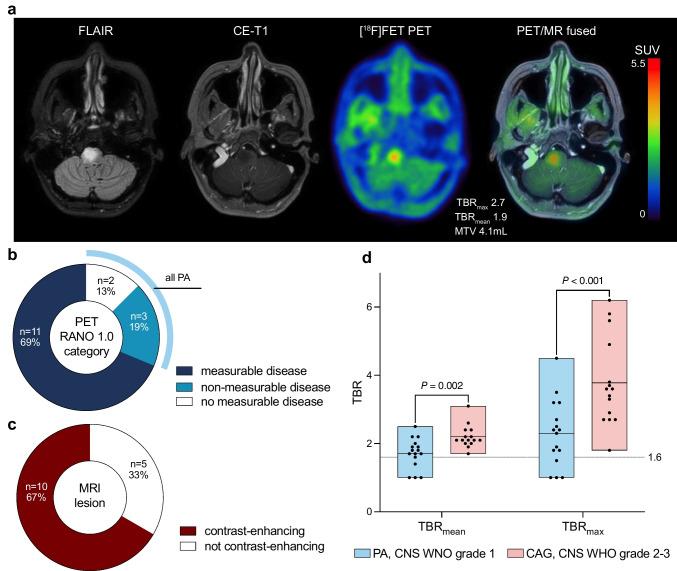
**a** MRI and [^18^F]FET PET of a 45-year-old female patient (#39) with a non-enhancing lesion on MRI and *measurable disease* on [^18^F]FET PET, diagnosed with high-grade astrocytoma with piloid features following stereotactic biopsy. **b** Distribution of [^18^F]FET uptake in treatment-naïve circumscribed astrocytic gliomas (CAG) stratified by PET RANO 1.0 categories and **c** contrast-enhancement on MRI. Of note, only pilocytic astrocytomas (PA) were classified as no or non-measurable disease according to PET RANO 1.0 categories. **d** Box plots illustrating significantly higher mean and maximum tumor-to-brain ratio (TBRs) in active CNS WHO grade 2–3 CAG compared to PA, CNS WHO grade 1

Among patients with untreated CAG, *measurable disease* was recorded in 69% of PET scans (11 out of 16 patients). All five patients without *measurable disease* had PA, resulting in a rate of 58% of patients with PA with *measurable disease* before diagnosis (7 out of 12 patients). Five patients with CAG presented without contrast-enhancing lesions before diagnosis (PA, *n* = 4; HGAP, *n* = 1). In one patient (#33), contrast-enhanced MRI was not performed. Of note, the patient with non-enhancing HGAP presented with *measurable disease* on [^18^F]FET PET and is presented as an illustrative example in Fig. 2a. PET RANO 1.0 read-outs and rates of contrast-enhancement of all untreated CAG are depicted in Figs. 2b and c. The comparison of active CNS WHO grades 2 or 3 to 1 CAG is shown in Fig. 2d, and detailed data for treatment-naïve patients are provided in Supplemental Table 2 (Online Resource 2). Among patients with serial PET assessed for response or those with PET to identify treatment-related changes, the PET RANO 1.0 criteria differed from MRI findings, assessed per the MRI RANO criteria, in five of 11 patients (45%).

### Assessment of treatment response

In eight patients, serial [^18^F]FET PET was performed to assess the response to 11 treatments (median time between PET scans, 5.7 months). Four of these 11 treatments (36%) consisted of alkylating chemotherapy with or without radiotherapy, and three treatments (27%) were BRAF-/MEK-directed targeted therapy with dabrafenib plus trametinib. Details on pretreatment TBR, PET RANO 1.0 response categories, and therapy are provided in Supplemental Table 3 (Online Resource 2).

According to the PET RANO 1.0 criteria, *PET-based partial response* was confirmed in three and complete response in one patient. In patients #37, #38, and #39 with PXA and HGAP evaluated for response to temozolomide or chemoradiation with temozolomide followed by maintenance temozolomide or lomustine and *PET-based partial response*, clinicoradiological follow-up over two, five, and six months, respectively, confirmed stable course (follow-up in all three patients ongoing at data collection).

**Fig. 3:**
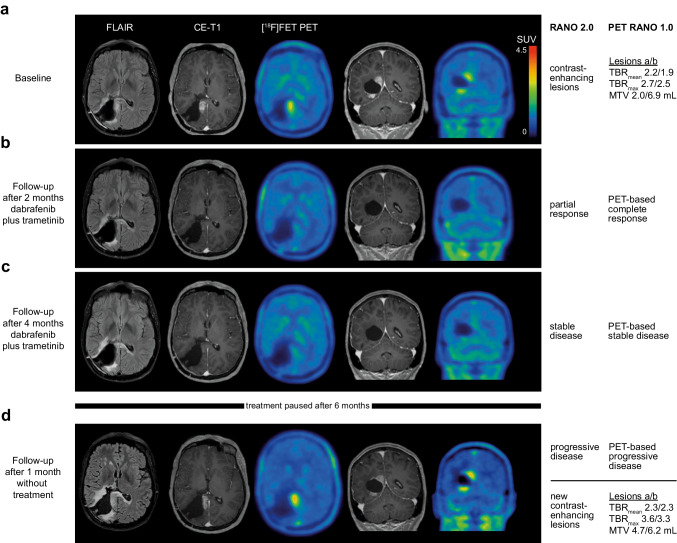
A 39-year-old woman was treated for an anaplastic pleomorphic xanthoastrocytoma (CNS WHO grade 3, BRAF^V600E^ mutated) adjacent to the right posterior ventricle. After first-line therapy consisting of radiotherapy and the second cycle of maintenance temozolomide, MRI revealed new enhancing lesions at the rim of the resection cavity, and [^18^F]FET PET confirmed a metabolically active tumor in spatial correlation to the enhancing lesions on MRI **a**. The relapse prompted second-line therapy with dabrafenib and trametinib. After two months of targeted therapy, enhancing lesions disappeared with remaining non-measurable disease on MRI (*Partial Response* according to the RANO 2.0 criteria) compared to the baseline MRI scan, while PET scan at the follow-up showed no [^18^F]FET uptake (*PET-based Complete Response* according to the PET RANO 1.0 criteria) **b**. At the second follow-up after 4 months, [^18^F]FET PET confirmed a durable response to targeted therapy with *PET-based Stable Disease* with no [^18^F]FET uptake **c**. Treatment-related toxicity necessitated discontinuation of dabrafenib and trametinib after a total of 6 months. After one month without treatment, epileptic seizures prompted neuroimaging, revealing *Progressive Disease* on MRI and two new measurable lesions on [^18^F]FET PET confirmed *PET-based Progressive Disease* according to the PET RANO 1.0 criteria **d**

In patient #4, who was diagnosed with PXA CNS WHO grade 3 and a BRAF^V600E^ mutation, [^18^F]FET PET identified *PET-based complete response* after two months of targeted therapy with dabrafenib plus trametinib and *PET-based progressive disease* after discontinuation of targeted treatment. MR-based response and progression were concordant with PET-based response categories. PET and MR images are presented in Fig. 3. Another patient (#42) with a BRAF^V600E^ mutation was evaluated for response to dabrafenib plus trametinib as a third-line treatment on two separate occasions, with confirmed treatment-related changes in both cases. During targeted therapy, *PET-based stable disease* was observed, while MRI showed progressive disease, and biopsy subsequently confirmed treatment-related changes. On another occasion, however, PET monitoring indicated progressive disease, yet the patient experienced a stable clinical course over seven months and was later diagnosed with treatment-related changes.

Three patients (27%) were evaluated for response to brachytherapy (#14, #34, and #42) with a median time between baseline and follow-up PET of 15 months (range, 5—16 months). In patient #14 with PA, *PET-based stable disease* and *partial response* on MRI were followed by more than 55 months of clinical and radiological stability. In patient #34 with HGAP, relapse was correctly confirmed neuropathologically after *PET-based progressive disease* and MRI progression. In contrast, patient #42 with PXA experienced relapse five months after *stable disease* on both PET and MRI.

### Treatment-related changes

**Fig. 4:**
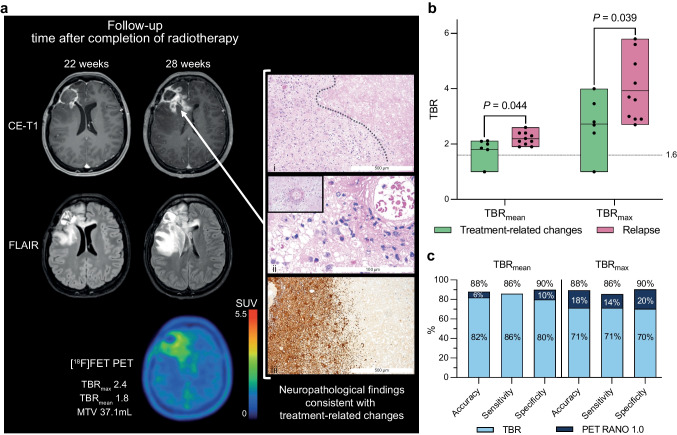
**a** MRI and [^18^F]FET PET of a 34-year-old female patient (#6) with astroblastoma, *MN1*-altered treated with radiotherapy at first relapse. Six months after radiotherapy, contrast-enhanced and FLAIR-weighted MRI suggested tumor relapse (middle column). In contrast, PET revealed [^18^F]FET uptake with tumor-to-brain ratios indicating treatment-related changes. Neuropathological evaluation of tissue obtained by re-resection confirmed treatment-related changes with predominantly (i) reactive and necrotic changes (dotted line between necrosis and reactive brain parenchyma; hematoxylin and eosin (H&E) staining; original magnification, 40x; scale bar, 500 μm), (ii) perivascular infiltration of inflammatory immune cells and fibrinoid vascular changes (ii; H&E staining; original magnification, 100x; scale bar, 100 μm), and (iii) reactive gliosis (immunohistochemistry with anti-GFAP staining; original magnification, 40x; scale bar, 500 μm). At the last follow-up, the patient was in a stable clinical condition after 3.3 years without relapse. **b** Box-plots illustrating significantly higher mean and maximum tumor-to-brain ratio (TBRs) in confirmed relapsed CNS WHO grades 2–3 CAG compared to treatment-related changes. **c** Bar plot illustrating the increase of up to 20% in accuracy, sensitivity, and specificity for identifying treatment-related changes after adding the PET RANO 1.0 criteria to TBRs

Treatment-related changes were diagnosed in 14 of 31 (45%) patients with MRI findings suggestive of tumor relapse who underwent additional [^18^F]FET PET (Supplemental Table 4; Online Resource 2). The confirmation of treatment-related changes was based on clinicoradiological follow-up in 9 cases (64%) or neuropathological evaluation in 5 cases (36%). Most treatment-related changes (71%, *n* = 10) were diagnosed following first-line therapy; across the cohort, the treatment line at the time of treatment-related changes diagnosis ranged from first to seventh. Treatments that preceded treatment-related changes included resection, stereotactic radiosurgery, brachytherapy, external beam radiotherapy alone or with concomitant and maintenance temozolomide chemotherapy, and BRAF-/MEK-targeted therapy with dabrafenib plus trametinib. Of 14 confirmed treatment-related changes, the diagnoses were PA (*n* = 8), PXA (*n* = 4), HGAP (*n* = 1), and AB (*n* = 1). In patient #42, treatment-related changes were confirmed in four biopsy-derived specimens throughout the treatment. A patient example is presented in Fig. 4a.

Based on the high rate of PA without *measurable disease* on PET (35%), the diagnostic performance of [^18^F]FET PET was evaluated only for CAG with CNS WHO grades 2–3. TBR_max_ and TBR_mean_ values of [^18^F]FET uptake (Fig. 4b) were significantly lower in treatment-related changes compared tumor relapses (TBR_max_, 2.7 ± 1.0 vs. 4.0 ± 1.2, *P* = 0.036; TBR_mean_, 1.8 ± 0.4 vs. 2.2 ± 0.2, *P* = 0.027). ROC analysis revealed that the optimal cut-off value of TBR_max_ for the identification of treatment-related changes was 3.6 (AUC, 0.84 ± 0.10; *P* = 0.022), with an accuracy of 71%; (sensitivity, 71%; specificity, 70%; positive predictive value (PPV), 63%; negative predictive value (NPV), 78%). For TBR_mean_, the optimal cut-off value was 2.1 (AUC, 0.81 ± 0.11; *P* = 0.032), with an accuracy of 82% (sensitivity, 86%; specificity, 80%; PPV, 75%; NPV, 89%).

As a second step, TBR values were combined with the PET RANO 1.0 criteria in patients for whom an earlier PET scan was available (*n* = 7). When the PET RANO 1.0 criteria were applied to these patients, with *PET-based progressive disease* indicating a true relapse, 7 out of 7 cases were correctly identified. As shown in Fig. 4c, adding the PET RANO 1.0 criteria (when applicable) to the TBR values improved the entire cohort’s accuracy, sensitivity, and specificity. In this approach, the PET RANO 1.0 response category *PET-based progressive disease* overruled threshold-based analyses of a single PET scan. For TBR_max_, the addition of the PET RANO 1.0 criteria resulted in an increase in accuracy by 18% (accuracy, 88%; sensitivity, 86%; specificity, 90%; PPV, 86%; NPV, 90%; *P* = 0.004). Adding the PET RANO 1.0 criteria to TBR_mean_ increased the accuracy by 6% (accuracy, 88%; sensitivity, 86%; specificity, 90%; PPV, 86%; NPV, 90%; *P* = 0.004).

## Discussion

This study suggests a potential of [^18^F]FET PET for addressing several clinical questions at different stages of diagnosing and treating patients with CAG. Although literature on PET imaging in adults with PA and CAG is limited, advanced imaging techniques such as PET are particularly valuable in this population. In line with its established utility throughout the diagnostic pathway in glioma, our data show that [^18^F]FET PET offers added value beyond MRI for the clinical management of adults with CAG, who generally have a worse prognosis than pediatric patients with CAG [23].

In our study, a substantial proportion of CAG had *measurable disease* on [^18^F]FET PET. Additionally, the 100% rate of measurable [^18^F]FET uptake in CAG CNS WHO grades 2–3 indicates the high sensitivity of amino acid PET for tumor detection. While MRI detects most circumscribed astrocytic gliomas, [^18^F]FET PET provides complementary metabolic information. The fact that [^18^F]FET PET detected all CAG CNS WHO grades 2–3 provides a basis for the use of PET regarding clinical questions in which amino acid PET has been shown its additive value in diffuse gliomas [24]. Consistent with prior initial reports [25], we observed higher TBRs in CAG CNS WHO grades 2–3 compared to PA CNS WHO grade 1. With approximately one-third of PA CNS WHO grade 1 presenting without *measurable disease*, PET imaging of a circumscribed lesion on MRI without measurable [^18^F]FET uptake does not rule out CAG but could align with PA CNS WHO grade 1 among other differential diagnoses.

Our results show that [^18^F]FET PET delineates metabolically active disease in CAG, including in non-enhancing lesions (e.g., Fig. 2a) and with higher uptake in CNS WHO grades 2–3, thereby providing information that may support surgical planning and guidance of tissue sampling; however, the present study did not prospectively evaluate operative decisions based on PET. Among the 16 untreated CAG cases, five tumors (PA, *n* = 4; HGAP, *n* = 1) lacked contrast enhancement on MRI (31%). Amino acid PET offers a key advantage in such cases, as tracer uptake occurs independently of blood–brain barrier disruption. This enables improved delineation of tumor extent, even in non-enhancing lesions, which may aid in identifying suitable biopsy targets and maximizing tissue yield for molecular diagnostics [26, 27]. Obtaining sufficient tissue for molecular analyses (> 80% tumor cells) is crucial, given the prevalence of targetable genetic alterations in CAG [9]. Direct prospective evidence that [^18^F]FET PET-guided biopsies improve diagnostic accuracy specifically in CAG is lacking. Although our study was not designed to assess biopsy accuracy directly, prior studies have demonstrated the value of amino acid PET in glioma delineation [27–29], including prospective evidence that [^18^F]FET PET identifies malignant tumor outside MRI contrast enhancement and effects biopsy results [30]. These findings combined with our results suggest a translational rationale for [^18^F]FET PET-guided biopsy targeting in circumscribed gliomas.

### Assessment of treatment response

We evaluated 22 [^18^F]FET PET scans conducted before and after 11 treatments using the PET RANO 1.0 criteria for response assessment. As depicted in Fig. 3, the application of the response criteria showed a *PET-based complete response* to treatment, *stable disease* during treatment, and *progressive disease* after treatment discontinuation. Given the heterogeneity of the cohort in terms of diagnoses and treatments and the small sample size, no statistical analysis was conducted regarding the prognostic value of a PET-based response. From the data in this study, we cannot claim clinical benefit of [^18^F]FET PET but view the results as hypothesis-generating. In this context, the proof of *measurable disease,* and the documentation of *PET-based partial* or *complete responses* and stable clinical courses over several months, indicate the general applicability of the method and criteria for response assessment, laying the foundation for further investigations. The PET RANO 1.0 criteria offer the first standardized response categories but have been proposed for diffuse gliomas and do not include CAG. Due to the rarity of CAG, prospective PET studies for response assessment are expected to encounter difficulties, such as limited accrual rates and variability in treatment protocols. Nevertheless, additional amino acid PET imaging should be encouraged in the clinical management of patients with CAG. Register studies, such as the EORTC-2013-BTG (GLIO-RARE) study, appear well-suited to validate our findings by providing a larger sample size and prospective data collection. To enable cross-study comparisons, we suggest that the next version of the PET RANO criteria should also address circumscribed gliomas.

The PET studies underlying the development for the PET RANO 1.0 criteria primarily focus on alkylating therapies in diffuse gliomas [21]. Targeted treatments such as BRAF-inhibitors (ESCAT evidence tier I) are increasingly being tested, used, and recommended [8, 9]. Evidence supporting the use of [^18^F]FET PET for response assessment in this context remains limited and largely case-based [31]. In our study, serial [^18^F]FET PET imaging detected metabolic responses to targeted therapies such as dabrafenib plus trametinib. These findings support our recommendation to consider extending the PET RANO 1.0 criteria to include CAG—pending validation of our results—as these tumors harbor targetable mutations more frequently compared to glioblastoma [9]. Given the anticipated expansion of targeted therapies in glioma, we advocate for prospective studies or registry-based evaluations to assess the applicability of the PET RANO criteria in this patient group. It should be noted that novel therapeutic agents, including targeted and immune-modulating treatments, could theoretically influence amino acid PET signals. This aspect has not been systematically evaluated and supports further investigation in future studies.

### Treatment-related changes

Lastly, we demonstrate the potential of [^18^F]FET PET in distinguishing between tumor relapse and treatment-related changes in patients with CNS WHO grades 2–3 CAG. Our findings indicate that a high diagnostic accuracy of 82% can be achieved. TBR_mean_ outperformed TBR_max_ in our study in terms of accuracy (82% vs. 71%), sensitivity (86% vs. 71%), and specificity (80% vs. 70%). In patients with both IDH-wildtype and IDH-mutant diffuse gliomas, amino acid PET using [^18^F]FET has shown high accuracy in identifying treatment-related changes ranging from 70 to 99% for TBRs alone [16, 32, 33]. A recent meta-analysis evaluating amino acid PET metrics in more than 1,000 patients with glioma reported pooled sensitivity and specificity of 83% and 79%, respectively [34]. To provide context, without implying cross-modality equivalence, a meta-analysis of MRI techniques in diffuse high-grade glioma reported pooled sensitivity/specificity of 90%/82% for diffusion-weighted imaging (DWI), 88%/88% for dynamic susceptibility contrast MRI (DSC-MRI), and 88%/77% for dynamic contrast-enhanced MRI (DCE-MRI) in distinguishing pseudoprogression from true progression [35]. These estimates pertain to diffuse high-grade gliomas; because our study comprises circumscribed astrocytic gliomas, we refrain from cross-tumor-class comparisons. [^18^F]FET PET has been evaluated in children and adolescents with brain tumors, including CAG [36–38]. It has to be mentioned that CAG are more common in children and adolescents compared to adult patients. To our knowledge, this series is the largest to evaluate [^18^F]FET PET for distinguishing treatment-related changes from tumor relapse in adult patients with CAG. The optimal threshold value for TBR_mean_ of 2.1 calculated in this study is well aligned with studies evaluating [^18^F]FET PET in patients with diffuse gliomas (range of TBR_mean_,1.8–2.1) [16, 32, 33]. However, the optimal cut-off value for TBR_max_ of 3.6 is higher than the usually reported cut-off values, which range from 1.9 to 3.5 [16, 32, 33, 39]. The ranges in thresholds reflect recalibrations of TBR value cut-offs across studies. With the PET RANO 1.0 criteria, recommendations now exist for standardized response assessment but not for identifying treatment-related changes [21]. Nevertheless, we applied the PET RANO 1.0 criteria in patients for whom earlier PET scans were available and demonstrated high diagnostic performance to identify treatment-related changes in patients with CAG. It should be emphasized that this analysis was based on PET RANO 1.0 response categories derived from serial PET scans, rather than classifications of measurable or non-measurable disease from a single scan using the thresholds for TBR_max_ and metabolic tumor volume. Our findings underscore the value of serial PET imaging and the need to validate the PET RANO 1.0 criteria and explore their potential for identifying treatment-related changes using the clear cut-offs of *PET-based progression*. It is prudent to note that the analyses regarding identification of treatment-related changes may have been susceptible to verification bias in cases where the reference standard was clinico-radiological follow-up that may have incorporated PET. Although PET thresholds/PET-RANO categories were applied retrospectively for this study and were not used prospectively (i.e., at the time of clinical management), we cannot fully exclude circularity. These findings should therefore be considered hypothesis-generating and require prospective validation with predefined histopathologic or rigorously blinded reference standards.

Addressing the cost-effectiveness of additional [^18^F]FET PET is challenging, as costs vary across countries and healthcare systems. Nevertheless, previous studies have demonstrated the cost-effectiveness of [^18^F]FET PET in patients with diffuse gliomas. For example, response assessment in glioma patients undergoing adjuvant temozolomide chemotherapy using [^18^F]FET PET has been shown to be cost-effective [40]. However, extrapolating these findings to CAG remains speculative and would require further investigation. Given the high costs of targeted therapies, PET-based treatment monitoring in CAG may be justified at reasonable cost.

This study has limitations given the retrospective design. Subgroups were small and heterogeneous, limiting power and generalizability. The number of neuropathological confirmation of treatment-related changes or relapse was not available in all, but only 16 patients, including 5 patients with confirmed treatment-related changes. It has to be pointed out that in the context of post-treatment gliomas, clinical-radiological follow-up is an accepted reference standard, and pathology is not invariably superior. Small stereotactic samples in treated gliomas face sampling error due to intratumoral heterogeneity and admixture of viable tumor with treatment-effect tissue; even expert neuropathology can misclassify when viable tumor is sparse or spatially focal. We did not adjust for multiple testing given the the hypothesis-generating design, increasing type I error risk. Selection bias is possible, as PET was obtained when clinical uncertainty was high. Operative decisions were not prospectively standardized or PET-driven, and histopathologic confirmation of PET “hot spots” was limited, precluding conclusions on biopsy accuracy or biologic extent. Imaging protocols and timing were not fully uniform and neuropathological analyses decentralized. Accordingly, findings should be interpreted as hypothesis-generating. However, it currently represents the largest series of patients with CAG classified per WHO CNS5 assessed with PET imaging across three high-volume centers. Moreover, the group of CAG includes tumors with biologically diverse profiles that extend beyond CNS WHO grading alone. In this context, data on differences in amino acid PET tracer uptake across these distinct biological subtypes, irrespective of targetable mutations, remain scarce. This warrants further investigation in future studies. Larger register studies such as the EORTC-2013-BTG (GLIO-RARE) study, appear particularly well-suited to validate our findings by providing a larger sample size and prospective data collection.

In summary, our results suggest added clinical value of [^18^F]FET PET in patients with adult circumscribed astrocytic gliomas. With the exception of PA, amino acid PET demonstrates high diagnostic performance in CAG imaging and results comparable to those observed in diffuse gliomas. The combination of TBR values with the PET RANO 1.0 criteria may represent a valuable tool to address the clinical challenge of treatment-related changes, with the potential to reduce overtreatment or premature discontinuation of treatment. Therefore, additional [^18^F]FET PET imaging should be considered in patients with CAG and equivocal MRI findings. The utility of [^18^F]FET PET for response assessment remains to be evaluated, but its clinical use and inclusion in registries seem warranted.

## Supplementary Information

Below is the link to the electronic supplementary material.

Supplementary file1 (PDF 95 KB)

Supplementary file2 (PDF 196 KB)

Supplementary file3 (PDF 113 KB)

## Data Availability

Data from this project may be shared at reasonable request to the corresponding author after regulatory approvals by ethics committees and competent authorities as well as signed data access agreements with all participating institution.
