# Supplementary material for: Clinical potential of [18F]FET PET in patients with circumscribed astrocytic glioma
Source: Eur J Nucl Med Mol Imaging. 2025 Nov 18;53(4):2764–76. doi: 10.1007/s00259-025-07654-9 (PMC12920724; doi:10.1007/s00259-025-07654-9)
Supplement: Supplementary file 1 — Supplementary file1 (PDF 95 KB) [file 259_2025_7654_MOESM1_ESM.pdf]

Clinical potential of [^{18}F]FET PET in patients with circumscribed astrocytic glioma

European Journal of Nuclear Medicine and Molecular Imaging

Jan-Michael Werner[1,2,3], Maximilian J. Mair[1,2,4], Michael M. Wollring[3], Enio Barci[4], Isabelle Stetter[3], Hannah C. Puhr[1,2], Caroline Tscherpel[5,6], Gabriele Stoffels[6], Johannes A. Hainfellner[7], Anna S. Berghoff[1,2], Vincent Sunder-Plassmann[1,2], Georg Widhalm[8], Franziska Eckert[9], Gregor Kasprian[10,11], Thomas S. Nakuz[10,12], Alexander Beck[13], Patrick N. Harter[13,14,15], Louisa von Baumgarten[14,15,16,17], Niklas Thon[17,18], Stephan Schönecker[19], Robert Forbrig[20], Felix M. Mottaghy[21,22,23], Philipp Lohmann[6,21], Gereon R. Fink[3,6], Karl-Josef Langen[6,21,23], Norbert Galldiks[3,6,23], Nathalie L. Albert[4,15], and Matthias Preusser[1,2]

[1]Division of Oncology, [2]Christian Doppler Laboratory for Personalized Immunotherapy, Department of Medicine I, Medical University of Vienna, Vienna, Austria; [3]Dept. of Neurology, Faculty of Medicine and University Hospital Cologne, University of Cologne, Cologne, Germany; [4]Dept. of Nuclear Medicine, LMU University Hospital, LMU Munich, Munich, Germany; [5]Dept. of Neurology, University Hospital Frankfurt, Goethe University, Frankfurt am Main, Germany; [6]Inst. of Neuroscience and Medicine (INM-3, INM-4), Research Center Juelich, Juelich, Germany; [7]Division of Neuropathology and Neurochemistry, Department of Neurology, Medical University of Vienna, Vienna, Austria; [8]Dept. of Neurosurgery, Medical University of Vienna, Vienna, Austria; [9]Dept. of Radiation Oncology, Comprehensive Cancer Center Vienna, Medical University of Vienna, Vienna, Austria; [10]Dept. of Biomedical Imaging and Image-guided Therapy, Medical University of Vienna, Vienna, Austria; Divisions of [11]Neuroradiology and Musculoskeletal Radiology, [12]Nuclear Medicine, Medical University of Vienna, Vienna, Austria; [13]Center for Neuropathology and Prion Research, LMU University Hospital, LMU Munich, Munich, Germany; [14]German Cancer Consortium (DKTK), University Hospital, Partnersite LMU Munich, Munich, Germany; [15]Bavarian Cancer Research Center (BZKF), Munich, Germany; Depts. of [16]Neurology, [17]Neurosurgery, LMU University Hospital, LMU Munich, Munich, Germany; [18] Department of Neurosurgery, Knappschaft University Hospital Bochum, Bochum, Germany; [19]Dept. Radiation Oncology, LMU University Hospital, LMU Munich, Munich, Germany; [20]Institute of Neuroradiology, LMU University Hospital, LMU Munich, Munich, Germany; [21]Dept. of Nuclear Medicine, University Hospital RWTH Aachen, Aachen, Germany; [22]Department of Radiology and Nuclear Medicine, Maastricht University Medical Center (MUMC+), Maastricht, The Netherlands; [23]Center for Integrated Oncology Aachen Bonn Cologne Duesseldorf (CIO ABCD), Germany

Correspondence
Prof Matthias Preusser
Division of Oncology
Department of Medicine I
Medical University of Vienna
Vienna 1090, Austria
Phone: +43-(0)1-40400-44450
Email: matthias.preusser@meduniwien.ac.at

Supplementary Material (Online Resource 1)

SUPPLEMENTAL METHODS

PET Imaging

The tracer [^{18}F]FET was produced as described previously (*1,2*). Following the international practice guidelines for glioma imaging using PET with radiolabeled amino acids (*3*), all patients fasted for at least 4 hours before PET measurements. After intravenous injection of 2.5 - 3 MBq of [^{18}F]FET/kg of body weight, static (20-40 minutes post-injection) or dynamic PET images (0-40 and 0-50 minutes post-injection) were acquired using an ECAT EXACT HR+ PET scanner, Biograph PET/CT, or simultaneously with 3T MR imaging using a BrainPET insert (all PET scanners manufactured by Siemens, Erlangen, Germany). PET images were analyzed on a Hermes workstation (Hermes Medical Solutions; Stockholm Sweden) or PMOD Software (Bruker's Preclinical Imaging; Fällanden, Switzerland).

PET Data Analysis

Metabolic tumor volumes were assessed semi-automatically using a tumor-to-brain ratio (TBR) of > 1.6. The maximal and mean standardized uptake value (SUV) were evaluated as a ratio to the mean SUV of the healthy background (*4*), and served as measures for uptake intensity (TBR_{max} and TBR_{mean}). [^{18}F]FET PET scans were classified as having *no measurable* disease (absence of any visually increased signal in [^{18}F]FET PET), *non-measurable disease* (visible lesion with TBR_{max} < 1.6 or volume < 0.5 mL), or *measurable disease* (TBR_{max} > 1.6 and volume > 0.5 mL) according to PET RANO 1.0 criteria (*5*), and changes of [^{18}F]FET uptake for assessment of response were classified as *PET-based complete* or *partial response*, *PET-based stable disease*, or *PET-based progressive disease*.

Diagnosis of Tumor Progression and Treatment-related Changes

Supplementary Material (Online Resource 1)

The confirmation of tumor relapse was based on neuropathological analysis (i.e., presence of viable tumor tissue) after repeated biopsy or resection or confirmed clinicoradiologically if a neuropathological diagnosis was unavailable. For this, imaging or clinical worsening within six months after PET imaging prompting a change in treatment was considered tumor progression. Prominent necrosis with no or only minimal identifiable tumor remnants in neuropathological analysis confirmed treatment-related changes. Treatment-related changes were assumed if no treatment change was required for at least six months. These definitions, based on Young and colleagues (*6*), allowed a continued mild increase of enhancing lesions compared to the usual decrease or stabilization as long as no treatment change occurred during this period.

REFERENCES

1. Hamacher K, Coenen HH. Efficient routine production of the 18F-labelled amino acid O-2-18F fluoroethyl-L-tyrosine. *Appl Radiat Isot.* 2002;57:853-856.

2. Wester HJ, Herz M, Weber W, et al. Synthesis and radiopharmacology of O-(2-[18F]fluoroethyl)-L-tyrosine for tumor imaging. *J Nucl Med.* 1999;40:205-212.

3. Law I, Albert NL, Arbizu J, et al. Joint EANM/EANO/RANO practice guidelines/SNMMI procedure standards for imaging of gliomas using PET with radiolabelled amino acids and [(18)F]FDG: version 1.0. *Eur J Nucl Med Mol Imaging.* 2019;46:540-557.

4. Unterrainer M, Vettermann F, Brendel M, et al. Towards standardization of (18)F-FET PET imaging: do we need a consistent method of background activity assessment? *EJNMMI Res.* 2017;7:48.

5. Albert NL, Galldiks N, Ellingson BM, et al. PET-based response assessment criteria for diffuse gliomas (PET RANO 1.0): a report of the RANO group. *The Lancet Oncology.* 2024;25:e29-e41.

6. Young RJ, Gupta A, Shah AD, et al. Potential utility of conventional MRI signs in diagnosing pseudoprogression in glioblastoma. *Neurology.* 2011;76:1918-1924.
