# Supplementary material for: Clinical potential of [18F]FET PET in patients with circumscribed astrocytic glioma
Source: Eur J Nucl Med Mol Imaging. 2025 Nov 18;53(4):2764–76. doi: 10.1007/s00259-025-07654-9 (PMC12920724; doi:10.1007/s00259-025-07654-9)
Supplement: Supplementary file 2 — Supplementary file2 (PDF 196 KB) [file 259_2025_7654_MOESM2_ESM.pdf]

Clinical potential of [^{18}F]FET PET in patients with circumscribed astrocytic glioma

European Journal of Nuclear Medicine and Molecular Imaging

Jan-Michael Werner[1,2,3], Maximilian J. Mair[1,2,4], Michael M. Wollring[3], Enio Barci[4], Isabelle Stetter[3], Hannah C. Puhr[1,2], Caroline Tscherpel[5,6], Gabriele Stoffels[6], Johannes A. Hainfellner[7], Anna S. Berghoff[1,2], Vincent Sunder-Plassmann[1,2], Georg Widhalm[8], Franziska Eckert[9], Gregor Kasprian[10,11], Thomas S. Nakuz[10,12], Alexander Beck[13], Patrick N. Harter[13,14,15], Louisa von Baumgarten[14,15,16,17], Niklas Thon[17,18], Stephan Schönecker[19], Robert Forbrig[20], Felix M. Mottaghy[21,22,23], Philipp Lohmann[6,21], Gereon R. Fink[3,6], Karl-Josef Langen[6,21,23], Norbert Galldiks[3,6,23], Nathalie L. Albert[4,15], and Matthias Preusser[1,2]

[1]Division of Oncology, [2]Christian Doppler Laboratory for Personalized Immunotherapy, Department of Medicine I, Medical University of Vienna, Vienna, Austria; [3]Dept. of Neurology, Faculty of Medicine and University Hospital Cologne, University of Cologne, Cologne, Germany; [4]Dept. of Nuclear Medicine, LMU University Hospital, LMU Munich, Munich, Germany; [5]Dept. of Neurology, University Hospital Frankfurt, Goethe University, Frankfurt am Main, Germany; [6]Inst. of Neuroscience and Medicine (INM-3, INM-4), Research Center Juelich, Juelich, Germany; [7]Division of Neuropathology and Neurochemistry, Department of Neurology, Medical University of Vienna, Vienna, Austria; [8]Dept. of Neurosurgery, Medical University of Vienna, Vienna, Austria; [9]Dept. of Radiation Oncology, Comprehensive Cancer Center Vienna, Medical University of Vienna, Vienna, Austria; [10]Dept. of Biomedical Imaging and Image-guided Therapy, Medical University of Vienna, Vienna, Austria; Divisions of [11]Neuroradiology and Musculoskeletal Radiology, [12]Nuclear Medicine, Medical University of Vienna, Vienna, Austria; [13]Center for Neuropathology and Prion Research, LMU University Hospital, LMU Munich, Munich, Germany; [14]German Cancer Consortium (DKTK), University Hospital, Partnersite LMU Munich, Munich, Germany; [15]Bavarian Cancer Research Center (BZKF), Munich, Germany; Depts. of [16]Neurology, [17]Neurosurgery, LMU University Hospital, LMU Munich, Munich, Germany; [18] Department of Neurosurgery, Knappschaft University Hospital Bochum, Bochum, Germany; [19]Dept. Radiation Oncology, LMU University Hospital, LMU Munich, Munich, Germany; [20]Institute of Neuroradiology, LMU University Hospital, LMU Munich, Munich, Germany; [21]Dept. of Nuclear Medicine, University Hospital RWTH Aachen, Aachen, Germany; [22]Department of Radiology and Nuclear Medicine, Maastricht University Medical Center (MUMC+), Maastricht, The Netherlands; [23]Center for Integrated Oncology Aachen Bonn Cologne Duesseldorf (CIO ABCD), Germany

Correspondence
Prof Matthias Preusser
Division of Oncology
Department of Medicine I
Medical University of Vienna
Vienna 1090, Austria
Phone: +43-(0)1-40400-44450
Email: matthias.preusser@meduniwien.ac.at

Supplementary Material (Online Resource 2)

Supplemental Table 1: Patient characteristics, number and indications for [^{18}F]FET PET

#	Sex, age (years) at initial diagnosis	Diagnosis WHO CNS 5	EoR at initial diagnosis	Molecular targets ESCAT I-III	Neurofibromatosis type 1 (germline mutation)	Number of [^{18}F]FET PET	Indications for [^{18}F]FET PET
**1**	F, 27	PA, grade 1	B	no	n.a.	1	diagnostic before initial diagnosis
**2**	F, 25	PXA, grade 3	GTR	BRAFv600e, PDGFRA	no	2	relapse vs treatment-related changes
**3**	M, 25	PA, grade 1	GTR	no	no	1	diagnostic before initial diagnosis
**4**	F, 39	PXA, grade 3	STR	BRAFv600e	no	4	response assessment
**5**	M, 20	PXA, grade 3	GTR	no	no	1	relapse vs treatment-related changes
**6**	F, 30	AB, *MN1*-altered	GTR	PDGFRA	no	1	relapse vs treatment-related changes
**7**	M, 27	PA, grade 1	STR	no	no	1	diagnostic before initial diagnosis
**8**	M, 19	PA, grade 1	B	KIAA1549::BRAF	n.a.	1	diagnostic before initial diagnosis
**9**	M, 71	HGAP	B	no	n.a.	1	diagnostic before initial diagnosis
**10**	M, 23	HGAP	B	no	n.a.	4	relapse vs treatment-related changes
**11**	F, 34	PA, grade 1	STR	no	n.a.	2	relapse vs treatment-related changes
**12**	F, 25	PXA, grade 3	GTR	BRAFv600e	n.a.	1	relapse vs treatment-related changes
**13**	M, 53	PA, grade 1	n.a.	NF1	yes	1	relapse vs treatment-related changes
**14**	F, 40	PA, grade 1	B	no	n.a.	2	diagnostic before initial diagnosis<br>response assessment
**15**	F, 27	PA, grade 1	B	n.a.	n.a.	1	relapse vs treatment-related changes
**16**	M, 17	PA, grade 1	B	no	n.a.	1	relapse vs treatment-related changes
**17**	F, 30	HGAP	B	NF1	n.a.	3	relapse vs treatment-related changes
**18**	F, 44	PA, grade 1	B	no	n.a.	1	relapse vs treatment-related changes
**19**	M, 14	PA, grade 1	n.a.	NF1	yes	1	relapse vs treatment-related changes
**20**	M, 56	PA, grade 1	STR	n.a.	n.a.	1	diagnostic before initial diagnosis
**21**	M, 52	PA, grade 1	GTR	n.a.	n.a.	1	relapse vs treatment-related changes
**22**	F, 35	PA, grade 1	STR	NF1	yes	1	relapse vs treatment-related changes
**23**	F, 35	PA, grade 1	B	no	n.a.	1	relapse vs treatment-related changes
**24**	F,14	PA, grade 1	B	no	n.a.	1	relapse vs treatment-related changes
**25**	M,16	PA, grade 1	B	n.a.	n.a.	1	relapse vs treatment-related changes
**26**	M, 30	PA, grade 1	STR	n.a.	n.a.	1	diagnostic before initial diagnosis
**27**	M, 64	HGAP	R	no	n.a.	1	relapse vs treatment-related changes
**28**	F, 50	PA, grade 1	B	NF1	yes	1	diagnostic before initial diagnosis
**29**	F, 24	PA, grade 1	B	KIAA1549::BRAF	no	1	diagnostic before initial diagnosis
**30**	F, 42	PA, grade 1	GTR	no	no	1	diagnostic before initial diagnosis
**31**	M, 19	PA, grade 1	STR	no	no	1	relapse vs treatment-related changes
**32**	F, 32	PXA, grade 2	n.a.	no	no	1	relapse vs treatment-related changes
**33**	F, 31	PA, grade 1	B	BRAFv600e	no	1	diagnostic before initial diagnosis
**34**	F, 47	HGAP	STR	no	no	4	response assessment<br>relapse vs treatment-related changes

Supplementary Material (Online Resource 2)

#	Sex, age (years) at initial diagnosis	Diagnosis WHO CNS 5	EoR at initial diagnosis	Molecular targets ESCAT I-III	Neurofibromatosis type 1 (germline mutation)	Number of [^{18}F]FET PET	Indications for [^{18}F]FET PET
**35**	M, 37	PA, grade 1	B	no	no	1	diagnostic before initial diagnosis
**36**	F, 73	PA, grade 1	n.a.	n.a.	no	3	relapse vs treatment-related changes
**37**	F, 70	PXA, grade 2	B	BRAFv600e	no	2	response assessment
**38**	M, 34	HGAP	B	no	no	4	diagnostic before initial diagnosis<br>response assessment<br>relapse vs treatment-related changes
**39**	F, 45	HGAP	B	no	no	2	diagnostic before initial diagnosis<br>response assessment
**40**	M, 19	PXA, grade 2	GTR	BRAFv600e	no	1	relapse vs treatment-related changes
**41**	F, 20	PXA, grade 2	GTR	no	no	1	diagnostic before initial diagnosis
**42**	M, 26	PXA, grade 3	B	BRAFv600e	no	17	response assessment<br>relapse vs treatment-related changes

Abbreviations: AB = astroblastoma; **APA =** anaplastic pilocytic astrocytoma; **B =** biopsy; **EoR =** extent of resection; **ESCAT =** ESMO Scale for Clinical Actionability of Molecular Targets; **F** = female; **GTR =** gross-total resection; **HGAP =** high-grade astrocytoma with piloid features; **M** = male; **meth** = MGMT promoter methylated; **NF1 =** neurofibromin 1; **n.a.** = not available; **PA =** pilocytic astrocytoma; **PDGFR =** platelet-derived growth factor receptor A; **PXA =** pleomorphic xanthoastrocytoma; **R =** resection without information of extent; **STR =** subtotal resection

Supplementary Material (Online Resource 2)

Supplemental Table 2: Overview of [18F]FET PET examinations in patients with treatment-naïve circumscribed astrocytic glioma

N	Pat #	MRI		[18F]FET PET				Time between PET and diagnosis (biopsy/resection)	Diagnosis and WHO CNS grade
		Number of lesions	CE	Number of lesions	TBR_{max} / TBR_{mean}	MTV (mL)	PET RANO 1.0 category		
1	1	1	no	1	1.8 / 1.7	0.1	non-measurable disease	8 weeks	PA, 1
2	3	1	no	0	n.m.	n.m.	no measurable disease	2 weeks	PA, 1
3	7	1	yes	1	2.7 / 1.9	5.0	measurable disease	1 day	PA, 1
4	8	1	no	0	n.m.	n.m.	no measurable disease	4 weeks	PA, 1
5	9	1	yes	1	6.2 / 3.1	31.0	measurable disease	1 week	HGAP, 3
6	14	1	yes	1	2.4 /1.6	37.1	measurable disease	6 years	PA, 1
7	20	1	yes	1	4.5 / 2.5	7.9	measurable disease	1 week	PA, 1
8	26	1	yes	1	3.5 / 2.2	1.2	measurable disease	7 weeks	PA, 1
9	28	1	yes	1	2.4 / 1.9	2.3	measurable disease	4 years	PA, 1
10	29	1	no	1	1.5 / 1.4	n.m.	non-measurable disease	3 weeks	PA, 1
11	30	1	yes	1	2.3 / 1.8	0.7	measurable disease	1 week	PA, 1
12	33	1	n.p.	1	1.8 / 1.7	0.4	non-measurable disease	1 week	PA, 1
13	35	1	yes	1	2.5 / 1.8	8.6	measurable disease	2 weeks	PA, 1
14	38	1	yes	1	2.2 / 1.8	2.2	measurable disease	2 days	HGAP, 3
15	39	1	no	1	2.7 / 1.9	4.1	measurable disease	2 weeks	HGAP, 3
16	41	1	yes	1	3.3 / 2.1	7.0	measurable disease	2 weeks	PXA, 2

Abbreviations: AB = astroblastoma; **CE =** contrast-enhancement; **F** = female; **HGAP =** high-grade astrocytoma with piloid features; **M** = male; **meth** = MGMT promoter methylated; **MD** = measurable disease according to PET RANO 1.0 criteria; **n.m.** = not measurable; **n.p. =** not performed; **PA =** pilocytic astrocytoma; **PXA =** pleomorphic xanthoastrocytoma; **TBR =** tumor-to-brain ratio of [18F]FET uptake

Supplementary Material (Online Resource 2)

Supplemental Table 3: [18F]FET PET for the assessment of treatment response

N	Pat #	Tumor type, WHO CNS grade	Line of treatment	Treatment evaluated for response	[18F]FET PET findings			MRI response	Follow-up / confirmation
					Baseline	Follow-up	PET RANO 1.0 response criteria		
**1**	**4**	PXA, 3	First line	dabrafenib plus trametinib	TBR_{max}, 2.7 / 2.5<br>TBR_{mean}, 2.2 / 1.9<br>MTV, 2.0 / 6.9 mL	no MD	PET-based complete response	partial response	Stable clinical and imaging (PET and MRI) follow-up for 5 months
**2**	**10**	HGAP	Third line	bev	TBR_{max}, 3.7 / 2.4<br>TBR_{mean}, 2.2 / 1.8<br>MTV, 1.0 / 1.3 mL	TBR_{max}, 3.4 / 2.9<br>TBR_{mean}, 2.1 / 1.1<br>MTV, 6.3 / 3.7 mL<br>New lesions<br>TBR_{max}, 4.2 / 3.1<br>TBR_{mean}, 2.3 / 2.1<br>MTV, 5.4 / 6.4 mL	PET-based progressive disease	progressive disease	The patient died 4 months after the follow-up PET
**3**	**14**	PA, 1	First line	I-125 BT	TBR_{max}, 2.3<br>TBR_{mean}, 1.8<br>MTV, 1.2 mL	TBR_{max}, 2.3<br>TBR_{mean}, 1.7<br>MTV, 0.7 mL	PET-based stable disease	partial response	Stable clinical and imaging follow-up >55 months
**4**	**34**	HGAP, 3	Second line	I-125 BT	TBR_{max}, 2.6<br>TBR_{mean}, 2.0<br>MTV, 1.6 mL	TBR_{max}, 2.1<br>TBR_{mean}, 1.8<br>MTV, 3.0 mL	PET-based progressive disease	progressive disease	Neuropathologically confirmed relapse
**5**	**34**	HGAP, 3	Third line	TMZ	TBR_{max}, 2.8<br>TBR_{mean}, 2.1<br>MTV, 4.0 mL	TBR_{max}, 2.9<br>TBR_{mean}, 2.1<br>MTV, 5.7 mL	PET-based stable disease	progressive disease	Radiotherapy, afterwards stable clinical and imaging follow-up for >30 months
**6**	**37**	PXA, 2	Third line	TMZ	TBR_{max}, 2.9 / 3.6<br>TBR_{mean}, 2.0 / 2.2<br>MTV, 4.4 / 4.9 mL	TBR_{max}, 2.4 / 2.9<br>TBR_{mean}, 1.8 / 2.0<br>MTV, 2.7 / 4.4 mL	PET-based partial response	stable disease	Lost to follow-up
**7**	**38**	HGAP	First line	TMZ-RCx and lom	TBR_{max}, 3.5<br>TBR_{mean}, 2.4<br>MTV, 6.3 mL	TBR_{max}, 2.5<br>TBR_{mean}, 1.8<br>MTV, 3.5 mL	PET-based partial response	partial response	Stable clinical and imaging follow-up (MRI) for >5 months
**8**	**39**	HGAP	First line	TMZ-RCx + TMZ	TBR_{max}, 2.7<br>TBR_{mean}, 1.9<br>MTV, 4.1 mL	TBR_{max}, 2.1<br>TBR_{mean}, 1.8<br>MTV, 2.3 mL	PET-based partial response	stable disease	Stable clinical and imaging follow-up (MRI) for >6 months
**9**	**42**	PXA, 3	First line	I-125 BT	TBR_{max}, 3.4<br>TBR_{mean}, 2.1<br>MTV, 4.2 mL	TBR_{max}, 3.2<br>TBR_{mean}, 2.2<br>MTV, 3.6 mL	PET-based stable disease	stable disease	Histopathological confirmed relapse after 5 months
**10**	**42**	PXA, 3	Third line	dabrafenib plus trametinib	TBR_{max}, 2.2<br>TBR_{mean}, 1.8<br>MTV, 4.7 mL	TBR_{max}, 2.7<br>TBR_{mean}, 2.0<br>MTV, 8.4 mL	PET-based progressive disease	stable disease	Histopathological confirmed treatment-related changes after 7 months
**11**	**42**	PXA, 3	Third line	dabrafenib plus trametinib	TBR_{max}, 2.8<br>TBR_{mean}, 1.9<br>MTV, 12.4 mL	TBR_{max}, 2.8<br>TBR_{mean}, 2.0<br>MTV, 10.9 mL	PET-based stable disease	progressive disease	Treatment-related changes in biopsy

Abbreviations: bev = bevacizumab; **HGAP =** high-grade astrocytoma with piloid features; **I-125 BT** = iodine-125 brachytherapy; **lom =** lomustine; **MD** = measurable disease according to PET RANO 1.0 criteria; **MTV =** metabolic tumor volume; **n.a.** = not available; **PA**

Supplementary Material (Online Resource 2)

= pilocytic astrocytoma; **PXA =** pleomorphic xanthoastrocytoma; **$TBR_{max/mean}$ =** maximum / mean tumor-to-brain ratio; **TMZ =** temozolomide; **TMZ-RCx =** radiotherapy with concomitant and maintenance temozolomide chemotherapy

Supplementary Material (Online Resource 2)

Supplemental Table 4: Overview of [18F]FET PET examinations for the differentiation of treatment-related changes from relapse

N	Pat #	Age (years) at PET	Treatment(s) before PET imaging	Time after last radiotherapy (months)	MRI findings indicating relapse	[18F]FET PET: TBR and tumor volume (MTV)	[18F]FET PET: PET RANO 1.0 category	Confirmation	Final diagnosis
**1**	**2**	27	first treatment line : TMZ-RCx	13	increasing CE	TBR_{max}, 2.3<br>TBR_{mean}, 1.9<br>MTV, 0.4 mL	non-measurable disease	neuropath	treatment-related changes
**2**	**2**	30	4 treatment lines: TMZ-RCx, SRS, re-resection, dab+tram (ongoing)	36	increasing CE	TBR_{max}, 3.6<br>TBR_{mean}, 2.1<br>MTV, 7.8 mL	measurable disease	clinicorad	relapse
**3**	**5**	25	first treatment line: TMZ-RCx	73	increasing CE	TBR_{max}, 4.9<br>TBR_{mean}, 2.1<br>MTV, 14.5 mL	measurable disease	neuropath	relapse
**4**	**6**	34	2 treatment lines : TMZ-RCx, re-radiation	6	increasing CE	TBR_{max}, 2.4<br>TBR_{mean}, 1.8<br>MTV, 37.1 mL	measurable disease	neuropath	treatment-related changes
**5**	**10**	24	first treatment line: TMZ-RCx	7	increasing CE	Old lesion: TBR_{max}, 2.4; TBR_{mean}, 1.8; MTV, 1.3 mL<br>New lesion: TBR_{max}, 3.7; TBR_{mean}, 2.2; MTV, 1.0 mL	PET-based progressive disease	neuropath	relapse
**6**	**10**	25	2 treatment lines : TMZ-RCx, RT	2	New CE	New lesion: TBR_{max}, 4.2; TBR_{mean}, 2.3; MTV, 5.4 mL<br>New lesion: TBR_{max}, 3.1; TBR_{mean}, 2.0; MTV, 6.4 mL<br>Old lesion: TBR_{max}, 3.4 (+16%); TBR_{mean}, 2.1 (+43%; MTV, 6.3 mL (+395%)<br>Old lesion: TBR_{max}, 2.9 (-12%); TBR_{mean}, 1.9 (-21%); MTV, 3.7 mL (+448%)	PET-based progressive disease	clinicorad	relapse
**7**	**11**	35	first treatment line: RT	5	new CE lesion	TBR_{max}, 3.2<br>TBR_{mean}, 1.9<br>MTV, 19.8 mL	measurable disease	neuropath	relapse
**8**	**11**	36	first treatment line: RT	11	increasing CE	TBR_{max}, 3.2<br>TBR_{mean}, 2.0<br>MTV, 7.6 mL	measurable disease	clinicorad	relapse
**9**	**12**	52	2 treatment lines : re-resection, TMZ-RCx	86	increasing CE	TBR_{max}, 5.6<br>TBR_{mean}, 2.6<br>MTV, 29.2 mL	measurable disease	neuropath	relapse
**10**	**13**	53	first treatment line: resection	none	increasing CE	TBR_{max}, 3.3<br>TBR_{mean}, 2.0<br>MTV, 3.2 mL	measurable disease	neuropath	treatment-related changes
**11**	**15**	41	first treatment line: I-125 BT	16 years	increasing CE	TBR_{max}, 2.5<br>TBR_{mean}, 1.9<br>MTV, 2.9 mL	measurable disease	clinicorad	treatment-related changes
**12**	**16**	35	first treatment line: RT	17 years	increasing CE	TBR_{max}, 1.9<br>TBR_{mean}, 1.7<br>MTV, 1.0 mL	measurable disease	neuropath	relapse

Supplementary Material (Online Resource 2)

N	Pat #	Age (years) at PET	Treatment(s) before PET imaging	Time after last radiotherapy (months)	MRI findings indicating relapse	[^{18}F]FET PET			Confirmation	Final diagnosis
						TBR and tumor volume (MTV)		PET RANO 1.0 category		
**13**	**17**	32	first treatment line: TMZ-RCx	16	new CE	TBR$_{max}$, 4.0 (-5%)<br>TBR$_{mean}$, 2.1 (-2%)<br>MTV, 12.3 mL (-18%)		PET-based stable disease	clinicorad	treatment-related changes
**14**	**17**	33	first treatment line: TMZ-RCx	26	increasing CE	TBR$_{max}$, 3.8 (+4%)<br>TBR$_{mean}$, 2.1 (+4%)<br>MTV, 23.4 mL (+90%)	New lesion<br>TBR$_{max}$, 3.0<br>TBR$_{mean}$, 1.9<br>MTV, 6.9 mL	PET-based progressive disease	clinicorad	relapse
**15**	**18**	48	first treatment line: RT	16	increasing CE	TBR$_{max}$, 3.4<br>TBR$_{mean}$, 2.1<br>MTV, 5.4 mL		measurable disease	clinicorad	treatment-related changes
**16**	**19**	31	first treatment: resection	none	new CE	TBR$_{max}$, 2.7<br>TBR$_{mean}$, 2.0<br>MTV, 1.3 mL		measurable disease	clinicorad	treatment-related changes
**17**	**21**	59	first treatment: resection	none	new CE	no measurable disease		no measurable disease	neuropath	relapse
**18**	**22**	35	first treatment line: RT	2	increasing CE	TBR$_{max}$, 3.9<br>TBR$_{mean}$, 2.3<br>MTV, 26.8 mL		measurable disease	clinicorad	treatment-related changes
**19**	**23**	36	first treatment line: SRS	7	increasing CE	TBR$_{max}$, 3.5<br>TBR$_{mean}$, 2.2<br>MTV, 2.2 mL		measurable disease	clinicorad	treatment-related changes
**20**	**25**	18	first treatment line: TMZ-RCx	n.a.	increasing non-enhancing lesion	no measurable disease		no measurable disease	missing	n.a.
**21**	**27**	65	first treatment line: PT	1	increasing CE	TBR$_{max}$, 5.8<br>TBR$_{mean}$, 2.4<br>MTV, 11.4 mL		measurable disease	clinicorad	relapse
**22**	**31**	21	first treatment line: subtotal resection	none	new CE lesion	TBR$_{max}$, 1.8<br>TBR$_{mean}$, 1.7<br>MTV, 0.2 mL		non-measurable disease	clinicorad	treatment-related changes
**23**	**32**	48	3x resection	none	increasing CE	TBR$_{max}$, 1.8<br>TBR$_{mean}$, 1.7<br>MTV, 0.1 mL		non-measurable disease	neuropath	relapse
**24**	**34**	49	2 treatment lines: subtotal resection, I-125 BT	18	n.a.	TBR$_{max}$, 2.7 (+29%)<br>TBR$_{mean}$, 2.0 (+11%)<br>MTV, 3.5 mL (+17%)		PET-based progressive disease	neuropath	relapse
**25**	**36**	76	2 treatment lines: resection, I-125-BT	n.a.	n.a.	TBR$_{max}$, 3.2<br>TBR$_{mean}$, 2.2<br>MTV, 16.6 mL		measurable disease	neuropath	relapse
**26**	**36**	77	4 treatment lines: resection, I-125 BT,bev, RT	5	n.a.	TBR$_{max}$, 2.9<br>TBR$_{mean}$, 1.9<br>MTV, 48.7 mL		measurable disease	clinicorad	relapse
**27**	**38**	36	first treatment line: TMZ-RCx, lom,	19	new CE lesion	Old lesion<br>TBR$_{max}$, 2.8 (+12%)<br>TBR$_{mean}$, 2.5 (+47%)	New lesion<br>TBR$_{max}$, 2.9<br>TBR$_{mean}$, 2.4	PET-based progressive disease	neuropath	relapse

Supplementary Material (Online Resource 2)

N	Pat #	Age (years) at PET	Treatment(s) before PET imaging	Time after last radiotherapy (months)	MRI findings indicating relapse	[^{18}F]FET PET			Confirmation	Final diagnosis
						TBR and tumor volume (MTV)		PET RANO 1.0 category		
						MTV, 3.3 mL (-6%)	MTV, 0.8 mL			
**28**	**40**	22	first treatment line: resection	n.a.	increasing non-enhancing lesion	no measurable disease		no measurable disease	clinicorad	treatment-related changes
**29**	**42**	29	2 treatment lines: resection followed by I-125 BT, resection, dab+tram, (ongoing)	none	increasing CE	TBR_{max}, 2.8 (no change)<br>TBR_{mean}, 2.0 (+5%)<br>MTV, 10.9 mL -12%)		PET-based stable disease	neuropath	treatment-related changes
**30**	**42**	34	7 treatment lines: resection followed by I-125 BT, RT, dab+tram, bev, RT, re-resection followed by re-RT	7	increasing CE	TBR_{max}, 3.5<br>TBR_{mean}, 2.1<br>MTV, 30.1 mL		measurable disease	neuropath	treatment-related changes
**31**	**42**	36	7 treatment lines: resection followed by I-125 BT, RT, dab+tram, bev, RT, re-resection followed by re-RT, dab+tram (ongoing)	36	increasing cystic tumor areas	TBR_{max}, 2.7<br>TBR_{mean},1.9<br>MTV, 17.2 mL		measurable disease	neuropath	treatment-related changes

Abbreviations: bev = bevacizumab; **CE =** contrast-enhancement; **dab+tram =** dabrafenib plus trametinib; **I-125 BT** = iodine-125 brachytherapy; **lom =** lomustine; **meth** = MGMT promoter methylated; **MTV =** metabolic tumor volume; **n.a.** = not available; **PT =** proton therapy; **RT =** radiotherapy; **SRS =** stereotactic radiosurgery; **$TBR_{max/mean}$ =** maximum / mean tumor-to-brain ratio; **TMZ-RCx =** radiotherapy with concomitant and maintenance temozolomide chemotherapy
