# Supplementary material for: Clinical potential of [18F]FET PET in patients with circumscribed astrocytic glioma
Source: Eur J Nucl Med Mol Imaging. 2025 Nov 18;53(4):2764–76. doi: 10.1007/s00259-025-07654-9 (PMC12920724; doi:10.1007/s00259-025-07654-9)
Supplement: Supplementary file 3 — Supplementary file3 (PDF 113 KB) [file 259_2025_7654_MOESM3_ESM.pdf]

Clinical potential of [^{18}F]FET PET in patients with circumscribed astrocytic glioma

European Journal of Nuclear Medicine and Molecular Imaging

Jan-Michael Werner[1,2,3], Maximilian J. Mair[1,2,4], Michael M. Wollring[3], Enio Barci[4], Isabelle Stetter[3], Hannah C. Puhr[1,2], Caroline Tscherpel[5,6], Gabriele Stoffels[6], Johannes A. Hainfellner[7], Anna S. Berghoff[1,2], Vincent Sunder-Plassmann[1,2], Georg Widhalm[8], Franziska Eckert[9], Gregor Kasprian[10,11], Thomas S. Nakuz[10,12], Alexander Beck[13], Patrick N. Harter[13,14,15], Louisa von Baumgarten[14,15,16,17], Niklas Thon[17,18], Stephan Schönecker[19], Robert Forbrig[20], Felix M. Mottaghy[21,22,23], Philipp Lohmann[6,21], Gereon R. Fink[3,6], Karl-Josef Langen[6,21,23], Norbert Galldiks[3,6,23], Nathalie L. Albert[4,15], and Matthias Preusser[1,2]

[1]Division of Oncology, [2]Christian Doppler Laboratory for Personalized Immunotherapy, Department of Medicine I, Medical University of Vienna, Vienna, Austria; [3]Dept. of Neurology, Faculty of Medicine and University Hospital Cologne, University of Cologne, Cologne, Germany; [4]Dept. of Nuclear Medicine, LMU University Hospital, LMU Munich, Munich, Germany; [5]Dept. of Neurology, University Hospital Frankfurt, Goethe University, Frankfurt am Main, Germany; [6]Inst. of Neuroscience and Medicine (INM-3, INM-4), Research Center Juelich, Juelich, Germany; [7]Division of Neuropathology and Neurochemistry, Department of Neurology, Medical University of Vienna, Vienna, Austria; [8]Dept. of Neurosurgery, Medical University of Vienna, Vienna, Austria; [9]Dept. of Radiation Oncology, Comprehensive Cancer Center Vienna, Medical University of Vienna, Vienna, Austria; [10]Dept. of Biomedical Imaging and Image-guided Therapy, Medical University of Vienna, Vienna, Austria; Divisions of [11]Neuroradiology and Musculoskeletal Radiology, [12]Nuclear Medicine, Medical University of Vienna, Vienna, Austria; [13]Center for Neuropathology and Prion Research, LMU University Hospital, LMU Munich, Munich, Germany; [14]German Cancer Consortium (DKTK), University Hospital, Partnersite LMU Munich, Munich, Germany; [15]Bavarian Cancer Research Center (BZKF), Munich, Germany; Depts. of [16]Neurology, [17]Neurosurgery, LMU University Hospital, LMU Munich, Munich, Germany; [18] Department of Neurosurgery, Knappschaft University Hospital Bochum, Bochum, Germany; [19]Dept. Radiation Oncology, LMU University Hospital, LMU Munich, Munich, Germany; [20]Institute of Neuroradiology, LMU University Hospital, LMU Munich, Munich, Germany; [21]Dept. of Nuclear Medicine, University Hospital RWTH Aachen, Aachen, Germany; [22]Department of Radiology and Nuclear Medicine, Maastricht University Medical Center (MUMC+), Maastricht, The Netherlands; [23]Center for Integrated Oncology Aachen Bonn Cologne Duesseldorf (CIO ABCD), Germany

Correspondence
Prof Matthias Preusser
Division of Oncology
Department of Medicine I
Medical University of Vienna
Vienna 1090, Austria
Phone: +43-(0)1-40400-44450
Email: matthias.preusser@meduniwien.ac.at

Supplementary Material (Online Resource 3)

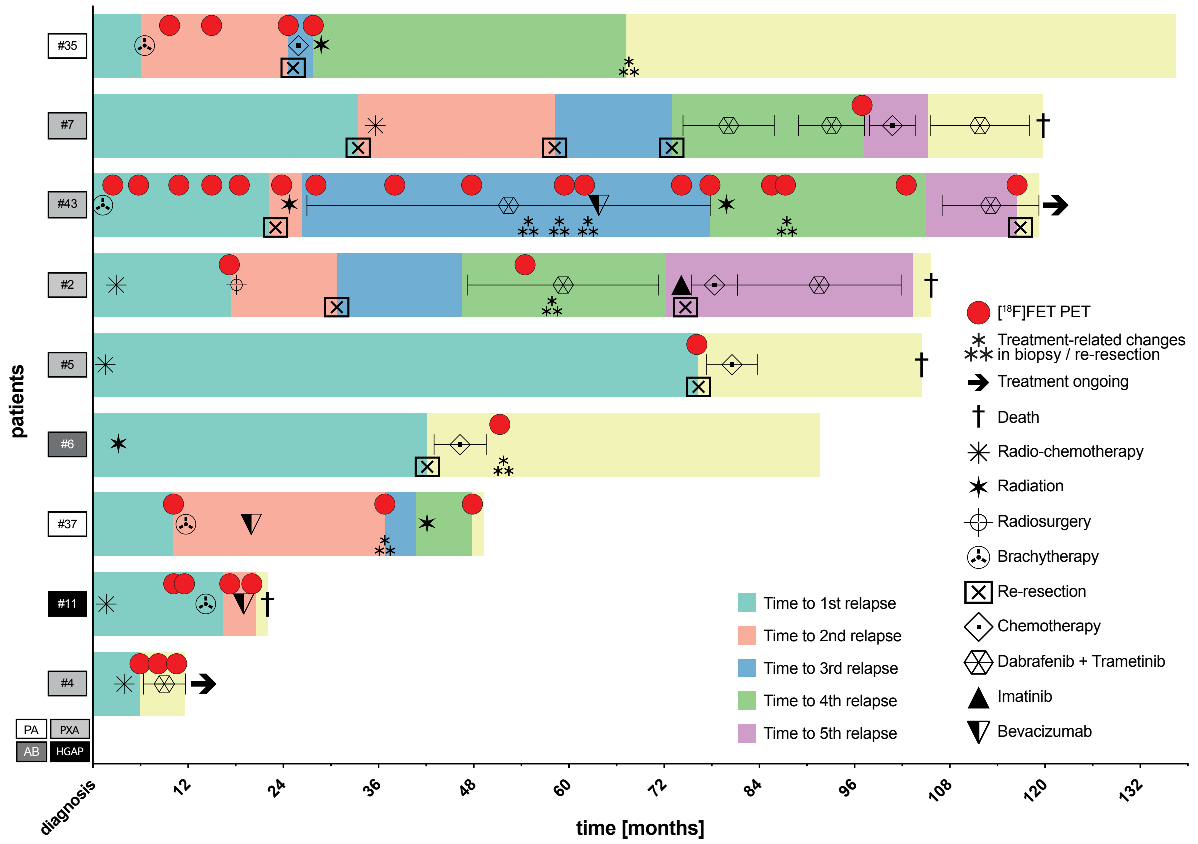

Supplemental Figure 1: Swimmer plot of eight selected patients after diagnosis of circumscribed astrocytic gliomas, including [^{18}F]FET PET scans at various time points throughout the disease course, highlighting the complexity and diversity of treatments. Patient bars are sorted by follow-up duration and color-coded based on times to relapses. Of note, MRI, PET, and tissue samples of patient #6 with astroblastoma and treatment-related changes are presented in Figure 4. **Abbreviations: AB** = astroblastoma; **HGAP =** high-grade astrocytoma with piloid features; **PA =** pilocytic astrocytoma; **PXA =** pleomorphic xanthoastrocytom
